# Effectiveness of formaldehyde in various soil types as a wide area decontamination approach for *Bacillus anthracis* spores

**DOI:** 10.1371/journal.pone.0277941

**Published:** 2022-11-18

**Authors:** William Richter, Michelle Sunderman, Zachary Willenberg, Michael Calfee, Shannon Serre, Joseph P. Wood

**Affiliations:** 1 Battelle Memorial Institute, Columbus, OH, United States of America; 2 US Environmental Protection Agency, Research Triangle Park, NC, United States of America; University of Connecticut, UNITED STATES

## Abstract

The purpose of this study was to evaluate and compare the decontamination efficacy of liquid formaldehyde solutions for three soil types (sand, loam, and clay) against spores of *Bacillus anthracis (B*.*a*.*)* and *Bacillus atrophaeus*. Approximately 1 x 10^8^ colony forming units were inoculated into each sample. Through a series of six bench-scale experiments, two concentrations and two volumes of liquid formaldehyde solution were then added to the soil samples and allowed to remain in contact for either 24 or 48 hours. Decontamination efficacy was assessed at either 22° or 10°C with or without lids atop the sample jars. Complete inactivation (no spores recovered from the soil samples, typically providing > 7 log reduction) of *B*.*a*. occurred in all soil types in five of the six tests, while complete inactivation of *B*. *atrophaeus* was achieved in all soil types for three of the six tests. The results demonstrated a higher probability of complete inactivation of spores for samples that were covered, samples that received the higher volume of formaldehyde, and those contaminated with *B*.*a*. Overall, the use of liquid formaldehyde solution (2.5–5%) was highly effective in inactivating entire spore populations (typically > 10^7^ CFU) for both *B*.*a*. and *B*. *atrophaeus* in the soil matrices studied. Covering the soil after application would allow for less formaldehyde solution to be used without impacting the overall efficacy of the process. The data from this study may aid in the selection of appropriate decontamination parameters when using liquid formaldehyde for soil remediation. The data may also aid in the decision to use *B*. *atrophaeus* as a surrogate for *B*.*a*. when performing further decontamination studies using liquid formalin solutions.

## Introduction

*Bacillus anthracis* (*B*.*a*.) is a highly infectious gram-positive, spore-forming bacteria and is the causative agent of anthrax disease. *B*.*a*. is categorized by the U.S. Centers for Disease Control and Prevention (CDC) as a Tier 1 select agent. Spore-forming biological agents, such as *B*.*a*., have been shown to survive outside their host following epizootic outbreaks for months to decades, which is largely due to their resistance to a broad range of environmental variables [1–6]. The CDC considers *B*.*a*. to be one of the most likely select agents to be used in an intentional biological attack and was in fact used in 2001 to contaminate several letters sent through the U.S. Postal Service [7]. Following a wide-area or a more site-specific contamination event from wild or domestic animals, many complex materials, including soil, may become contaminated, requiring extensive site remediation.

The ability to decontaminate soil contaminated with a biological agent such as *B*.*a*. is a highly complex challenge and is largely dependent on soil characteristics including organic content, chemical constituents, and physical properties such as density, particle sizes, and porosity [8]. Though limited, most literature related to the decontamination of soil matrices to date have focused on spore forming bacteria such as B.a. *and the use of liquid or fumigant applications*. Liquid decontaminants or sporicides offer ease of application as well as the ability to scale application rates to achieve various levels of penetration into the soil column. Several liquid sporicides have been previously shown to be effective for inactivation of various biological select agents on porous and non-porous building related surfaces [9–14]. However, when evaluated for the decontamination of soil, liquid decontaminants such as acidified chlorine bleach [15], peracetic acid [16], or aqueous chlorine dioxide [17] prove less effective. Other methods such as fumigants [18, 19] have shown high level surface efficacy but demonstrate challenges penetrating beyond the top 2–5 centimeters (cm) of soil. Lastly, dry thermal treatment was also shown to be effective in decontaminating soil contaminated with a *B*.*a*. surrogate, but efficacy varied by depth and other parameters [20].

Liquid formaldehyde solutions (5% in seawater) have previously been shown to be a relatively effective decontaminant after Gruinard Island was heavily contaminated with *B*.*a*. spores during World War II biological weapons testing from 1942 to 1943. Contamination distribution analysis showed that most *B*.*a*. spores, 40 years later, remained within the top 4 cm of soil. However, at the contamination point of origin, spores were found as deep as 50 cm. The 5% formaldehyde solution was applied by surface irrigation; however, copious amounts of the liquid were required (up to 50 liters per square meter [L/m^2^]) and in some heavily contaminated areas required repeated applications. Site remediation was successful as demonstrated by soil core sampling as well as a flock of sheep that was introduced to the island and allowed to graze for 5 months, none of which developed anthrax [21]. This is the only known and documented application of the use of formaldehyde for remediation of a site contaminated with *B*.*a*. spores.

The purpose of this evaluation was to study the efficacy of various concentrations of liquid formaldehyde solutions, over time, and optimize the volumes required to achieve high level efficacy using three soil types (clay, sand, and loam), in a controlled, laboratory setting. Both *B*.*a*. and *B*. *atrophaeus* spores were included and tested under the same conditions, side-by-side, to compare their resistance to inactivation by liquid formaldehyde in soil. The data developed from this study may aid in the decision to use *B*. *atrophaeus* as a surrogate for *B*. *a*. when performing further decontamination studies using liquid formalin solutions. Data related to the decontamination efficacy of both *B*.*a*. and *B*. *atrophaeus* are presented in terms of recovery of spores from positive control soil samples, log reduction (LR), and whether samples were completely inactivated (no spores detected).

## Materials and methods

### Test organisms

The virulent *B*.*a*. Ames spores used for this testing were prepared from qualified stocks at the Battelle Biomedical Research Center (BBRC, West Jefferson, OH) using a BioFlo 3000 fermenter (New Brunswick Scientific Co., Inc., Edison, NJ), as previously described (Rogers et al. 2005). Briefly, the spore lot was subjected to a stringent characterization and qualification process required by the Battelle standard operating procedure for spore production. Specifically, the spore lot was characterized prior to use by observation of colony morphology, direct microscopic observation of spore morphology, and size and determination of percent refractivity and percent encapsulation. In addition, the number of viable spores was determined by colony count and expressed as colony forming units per milliliter (CFU/mL). Theoretically, once plated onto bacterial growth media, each viable spore germinates and can yield one CFU, although spore clumping can mask total CFU determinations on growth medium. In addition, testing of the original spore lot was conducted for robustness of the Ames spores via hydrochloric acid resistance. The stock spore suspensions were prepared in sterile filtered water (SFW) at an approximate concentration of 1 x 10^9^ CFU/mL and stored at 5 ± 3°C, with the inoculum titer verified each day of testing.

The *B*. *atrophaeus* spores were supplied in powder form originally obtained from Dugway Proving Ground (Lot DJS-BG-004, Tooele County, UT). The *B*. *atrophaeus* stock spore suspensions were prepared in SFW at an approximate concentration of 1 x 10^9^ CFU/mL and stored at 5 ± 3°C. Genomic DNA was extracted from the spores and DNA genotype confirmed by polymerase chain reaction (matches ATCC 9372, Manassas, VA). In addition, the number of viable spores was determined by colony count and expressed as CFU/mL.

### Test materials

Three soil types were selected for testing (sand, clay, and loam) and obtained from Agvise Laboratories (Northwood, ND). These soil types were selected to represent a range of typical soil matrices. A full characterization of each soil type was performed by Agvise Laboratories to assess density, moisture content, and nutrients, as shown in S1 Table. Soil samples were prepared in 29.6 mL glass jars (Qorpak, Clinton, PA) by adding loose soil to a depth of one cm, to provide a total soil sample volume of 8 cm^3^. Due to the potential of soil natural flora confounding results, soil samples were sterilized by E-Beam sterilization (E-Beam Services, Lebanon, OH) prior to decontamination testing and sealed to preserve sterility until ready for use.

### Decontaminant

Formaldehyde (RICCA Chemical Company Cat. No. RSOF00104A, Arlington, TX) solutions were prepared in a chemical fume hood at the target concentration in advance of testing. The target concentration was obtained by diluting 37% formaldehyde (100% formalin) in SFW, then verified using the Technical Association for the Pulp Paper Industry (TAPPI) Method T600, analysis of formaldehyde in aqueous solutions titration method (TAPPI, Peachtree Corners, GA). Diluted formaldehyde solution was stored in a sealed container at ambient laboratory conditions prior to testing for up to one month.

### Sample processing and data collection

All work was conducted in a Biosafety Level (BSL-3) laboratory. For each soil type, three replicate soil samples were used at each contact time point. Samples were placed in a Class II Type A2 Biological Safety Cabinet (BSC; Baker Company, Sanford, ME) and inoculated with 10^8^ CFU via 100 μL (10 x 10 μL droplets) of the appropriate organism. The inoculum was dispersed throughout the soil sample by stirring, until homogenous, with a sterile inoculating loop (Fisher Cat. No. 22-363-607, Hampton, NH) and allowed to dry for one hour at ambient laboratory conditions. Following inoculation, the designated volume of formaldehyde at the appropriate concentration was added to the jars, stirred, and allowed to sit for the designated contact time and at the environmental conditions defined in Table 1. Sample jars were stored either in a Class III BSC or an incubator set to 10°C when target ambient air temperature was 22°C or 10°C, respectively. When tests were conducted at 10°C, the soil samples were stored in the incubator for a minimum of four hours prior to inoculation of spores. The lower temperature was chosen to represent mild winter conditions in the U.S.

**Table 1:** Overview of test matrix.

Test number	Target Temperature	Actual Temperature	Formaldehyde Concentration	Formaldehyde Volume (mL)	Contact Time (h)	Samples Sealed?
1	22°C	21°C	5%	2.8	48	No
2	22°C	22.8°C	2.5%	2.8	48	No
3	22°C	22.9°C	2.5%	2	48	No
4	22°C	23.5°C	2.5%	2	48	Yes
5	10°C	9.5°C	2.5%	2	48	Yes
6	10°C	9.7°C	2.5%	2	24	Yes

Ambient test conditions (target of 22°C) were not controlled during testing. All tests conducted with both species and all three soil types.

At the end of each time point, decontamination activity was neutralized by the addition of 9 mL Dey-Engley (DE) Broth (BD Cat. No., B4398318, Franklin Lakes, NJ). Samples were then agitated on an orbital shaker at 200 rotations per minute (RPM) for 15 minutes. For each soil type and experiment, three additional replicate samples were prepared as positive controls. Positive controls were treated with SFW, inoculated, allowed the same appropriate contact time, and extracted according to the procedure above. Additionally, one soil sample for each soil type was used as a blank (not inoculated) and included for each time point tested. The blank soil samples controlled for potential cross-contamination during testing.

To assess recovery of spores from soil samples, the resulting liquid extracts were serially diluted 10-fold in SFW. An aliquot (0.1 mL) of the selected dilutions and, when necessary, the undiluted extracts were plated onto Tryptic Soy Agar (BD Cat. No. 221283) plates in triplicate. The agar places were incubated for 21 ± 3 hours at 37 ± 2°C. Colonies were enumerated to determine survivorship and reduction of the viable spores following exposure.

### Statistical analysis

A binary response based on recovery of spores was the primary endpoint, e.g., a trial was recorded as a success if there was no recovery of spores (complete inactivation). (In the present study, since 10^8^ CFU were inoculated onto soil samples, complete inactivation of the samples represents a higher degree of decontamination than 6 LR.) A Firth logistic regression model was fitted to the full data set to test whether the proportions of successes were significantly associated with any effects included in the model. Five additional models of the same configuration were fit to all testing data, grouped by soil type (*i*.*e*., one model per soil type, three models) or organism (*i*.*e*., one model per organism, two models). The logistic regression model included main effects for formaldehyde concentration, formaldehyde volume, contact time, temperature, and sealed/unsealed samples. All statistical analyses were performed using SAS (version 9.4; Cary, NC). All results are reported at the 0.05 level of significance.

## Results

A total of six decontamination tests were conducted under various conditions, with each organism and soil type evaluated for every test. The mean amount of *B*.*a*. applied to each soil sample across all tests was 8.02 ± 0.18 log_10_ CFU, with a mean recovery of 7.20 ± 0.47 log_10_ CFU from control samples. The mean amount of *B*. *atrophaeus* applied to each soil sample across all tests was 8.11 ± 0.26 log_10_ CFU, with a mean recovery of 7.72 ± 0.31 log_10_ CFU from control samples. Recovery of spores of both species did not significantly vary by soil type. Significant effects at the 95% level of confidence were covered/uncovered, species, and formaldehyde volume, i.e., there was a higher probability of complete inactivation for samples that were covered, samples that received 2.8 mL formaldehyde, and for *B*.*a*. Ames, as shown in Table 2.

**Table 2:** Statistical analysis of ability to achieve complete inactivation.

Effect	Wald Test Statistic	p-value
Cover*	12.5564	0.0004
Concentration	2.4066	0.1208
Contact Time	1.7757	0.1827
Temperature	0.0000	1.0000
Volume*	12.2773	0.0005

Effects noted with an asterisk (*) are statistically significant at or below the 0.05 level.

### Formaldehyde concentration

**Fig 1:**
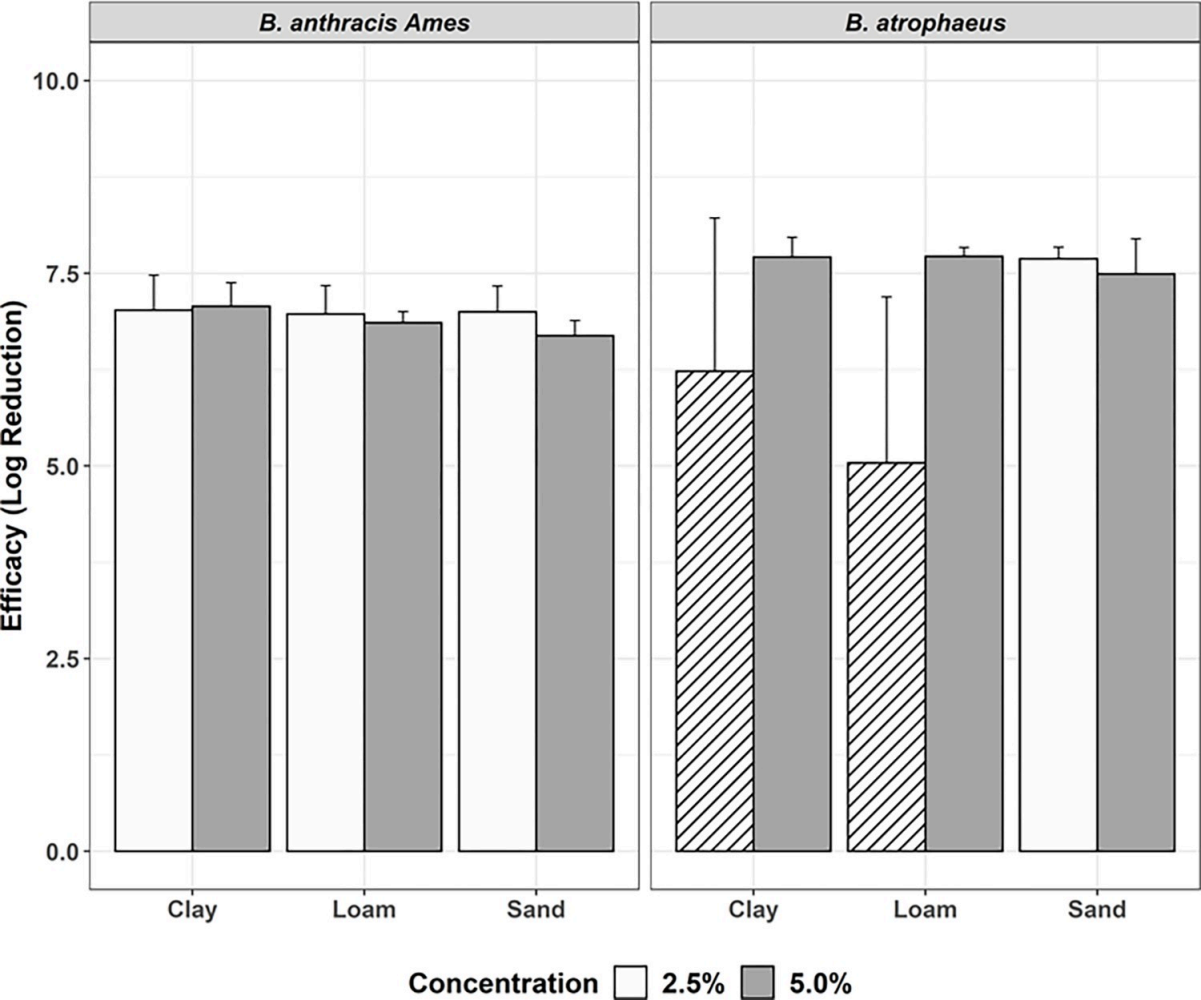
Efficacy of 2.5% (white; Test 2) vs. 5% (gray; Test 1) formaldehyde solutions. 2.8 mL, at ambient temperature, uncovered, against *B*.*a*. *and B*. *atrophaeus*, with efficacy represented by log reduction (± 95% CI) of bacteria, by soil type, and 48-hour contact time. Columns with stripes are samples that did not achieve complete inactivation.

Tests 1 and 2 evaluated the efficacy of formaldehyde at two concentrations, 5.0% and 2.5% (Fig 1), respectively. Samples were tested at ambient laboratory conditions (approximately 22°C) and were not sealed. The reduction in formaldehyde concentration did not result in reduced efficacy in sandy soil; complete inactivation was achieved with both concentrations and organisms. Efficacy was also not impacted for clay and loam soils inoculated with *B*.*a*. and both concentrations resulted in complete inactivation. For *B*. *atrophaeus*, efficacy was reduced somewhat in clay and loam soils when applied with 2.5% formaldehyde, resulting in incomplete inactivation with LR of 5.58 and 5.04, respectively. All subsequent tests were conducted with 2.5% formaldehyde. When data were evaluated for all tests, formaldehyde concentration did not have a statistically significant effect on whether a soil sample had complete inactivation (*P* = 0.121).

### Formaldehyde volume

**Fig 2:**
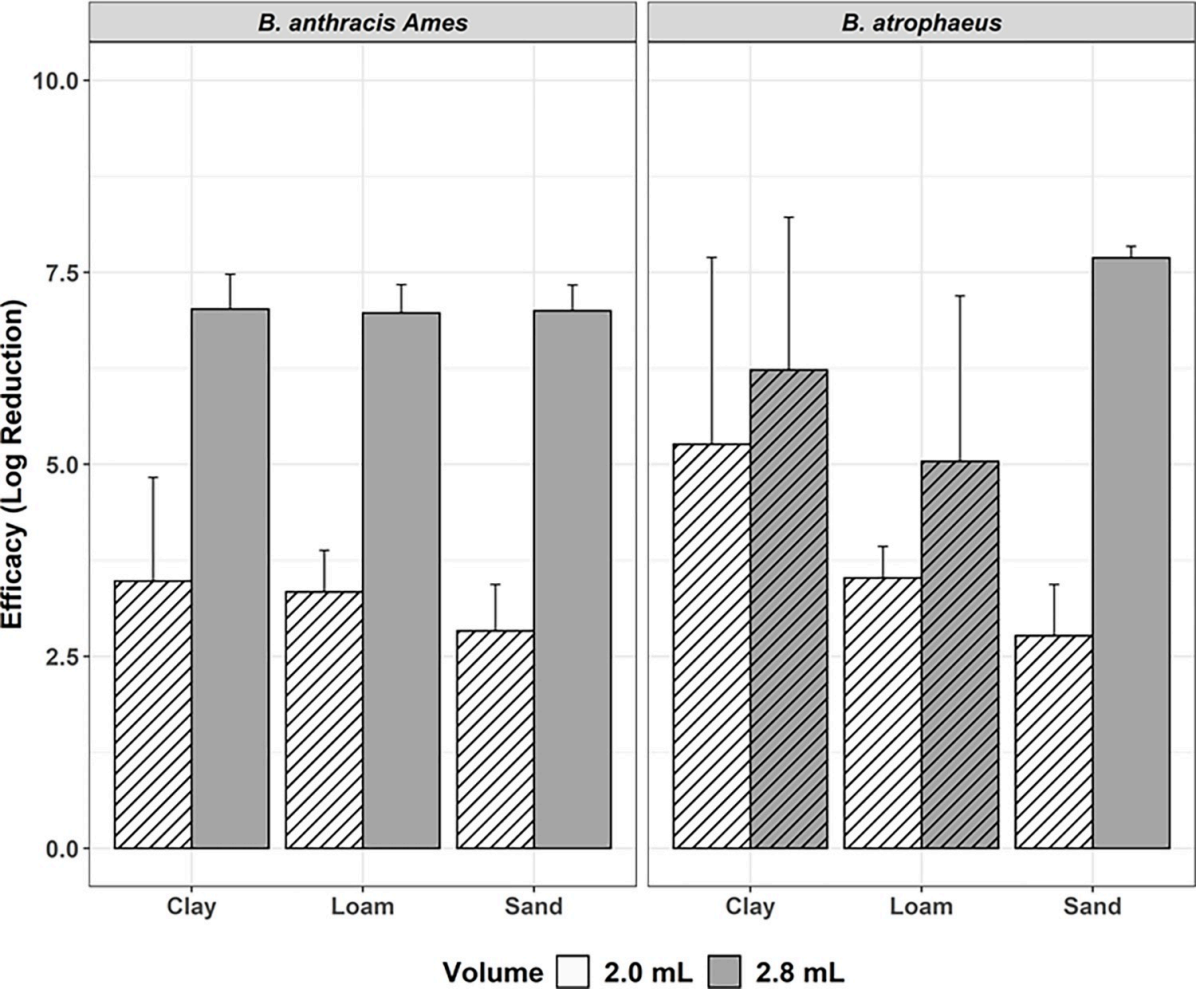
Efficacy of 2.0 mL (white; Test 3) vs. 2.8 mL (gray; Test 2) 2.5% formaldehyde. Tests conducted at ambient temperature, uncovered, against *B*.*a*. *and B*. *atrophaeus*, with efficacy represented by log reduction (± 95% CI) of bacteria, by soil type, and 48-hour contact time. Columns with stripes are samples that did not achieve complete inactivation.

Tests 2 and 3 evaluated the impact of formaldehyde volume on decontamination performance (Fig 2) at ambient laboratory conditions in unsealed samples. With a volume of 2.8 mL formaldehyde added to the 8 cm^3^ soil sample (Test 2), complete inactivation occurred in all *B*.*a*. samples and *B*. *atrophaeus* sand samples. Complete inactivation was not achieved in any soil type with the lower amount of formaldehyde solution added (Test 3), achieving an average reduction of 3.22 log_10_ for *B*.*a*. and an average reduction of 3.63 log_10_ for *B*. *atrophaeus*. The volume of formaldehyde solution added to the soil was determined to have a significant effect (*P* = 0.005).

### Covered/Sealed samples

**Fig 3:**
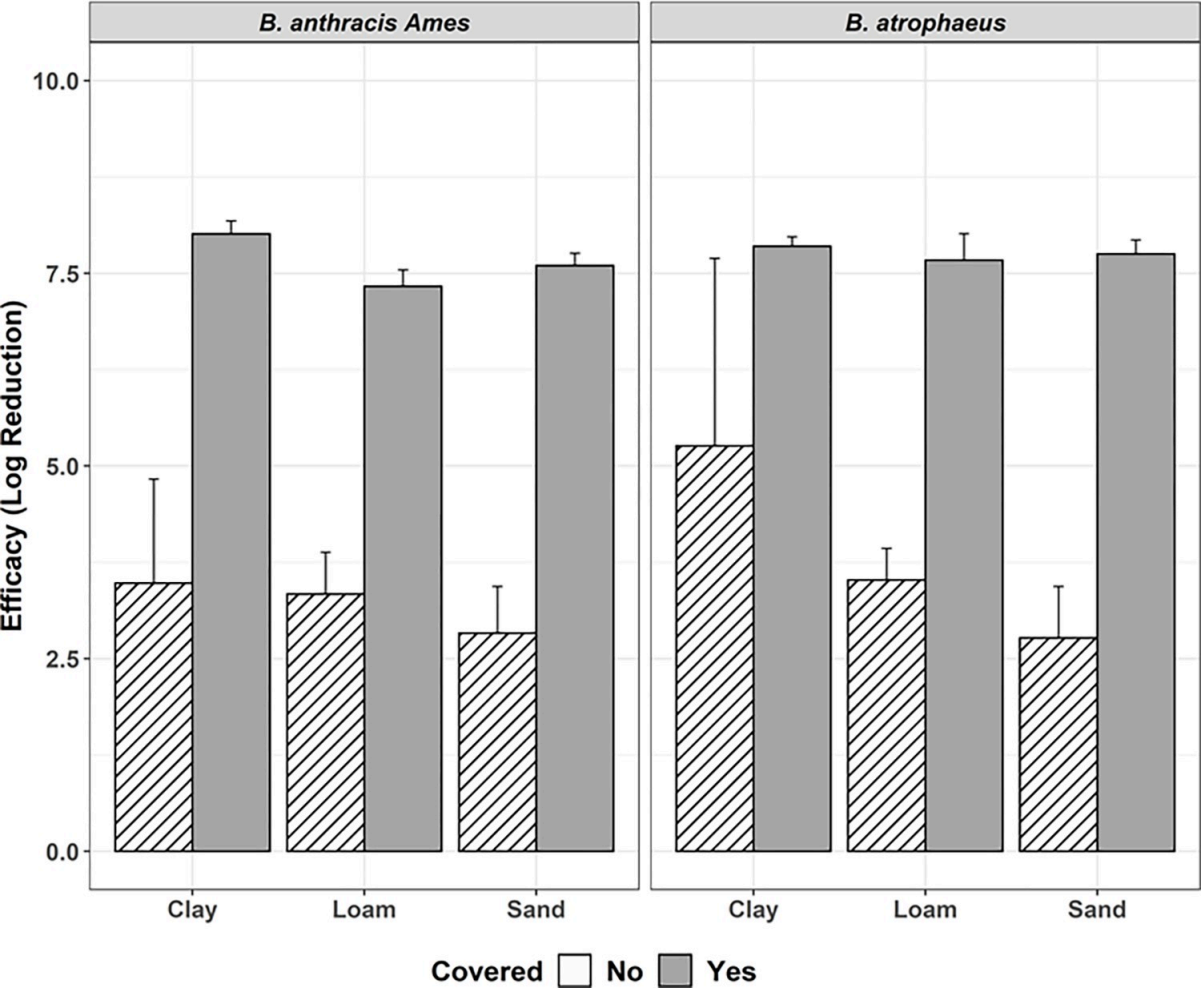
Efficacy formaldehyde with soil samples covered (gray; Test 4) vs. uncovered (white; Test 3). Tests conducted using 2 mL of 2.5% formaldehyde at ambient temperature, against *B*.*a*. *and B*. *atrophaeus*, with efficacy represented by log reduction (± 95% CI) of bacteria, by soil type, and 48-hour contact time. Columns with stripes are samples that did not achieve complete inactivation.

Tests 3 and 4 compared the effect of sealing the soil sample jars with lids on decontamination performance (Fig 3). Sealing the sample jars following the application of formaldehyde resulted in an increase in decontamination efficacy in Test 4, achieving complete inactivation in all samples. Log reduction values increased an average of 4.30 log_10_ when samples were sealed. Soil sample jars were sealed with lids for all following decontamination trials, and significantly increased decontamination efficacy (*P =* 0.0004).

### Temperature

**Fig 4:**
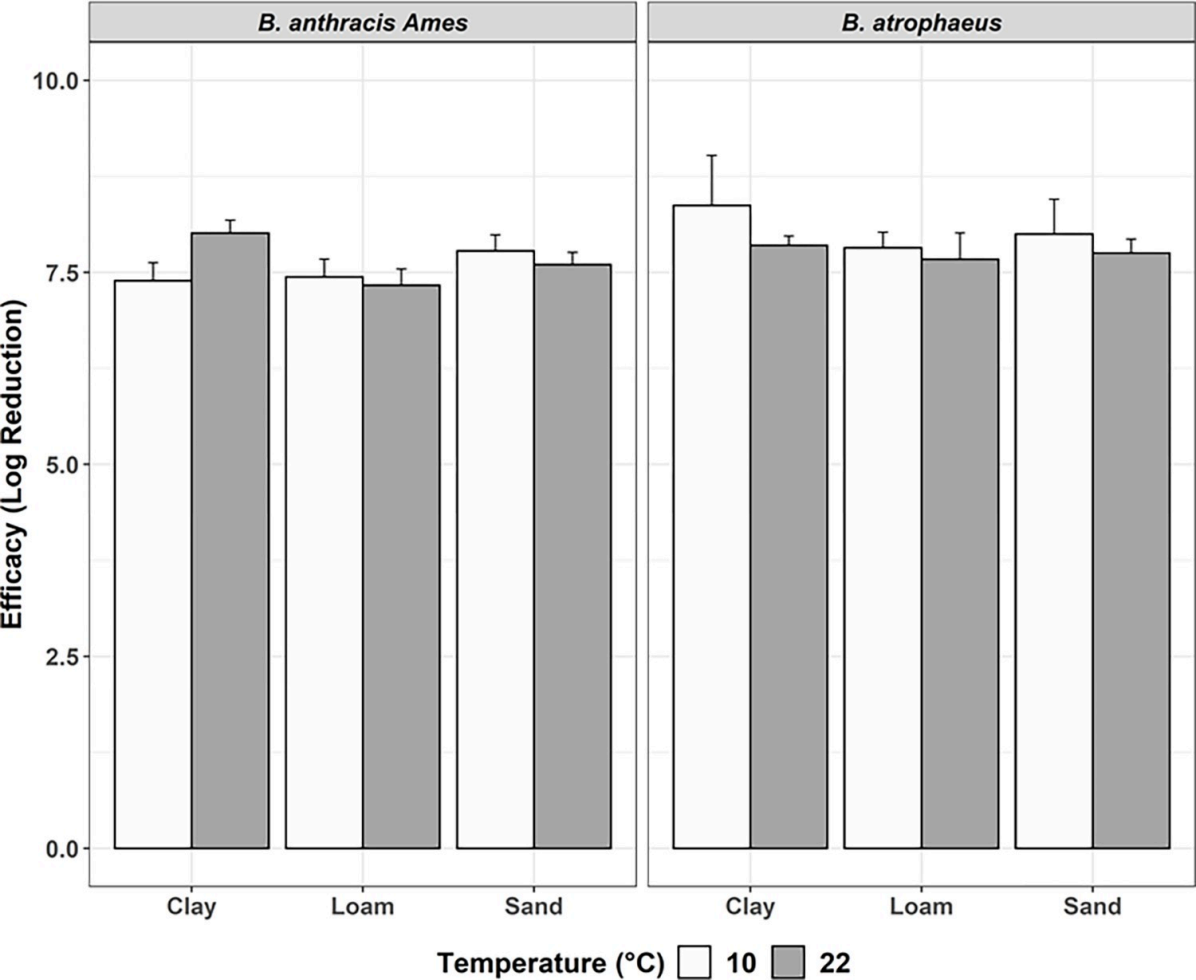
Efficacy of 2 mL of 2.5% formaldehyde, covered, at 10°C (white; Test 5) vs. 22°C (gray; Test 4). Tests conducted against *B*.*a*. *and B*. *atrophaeus*, with efficacy represented by log reduction (± 95% CI) of bacteria, by soil type, and 48-hour contact time. Columns with stripes are samples that did not achieve complete inactivation.

Tests 4 and 5 evaluated the effect of reducing temperature on formaldehyde efficacy in soil (Fig 4). While all previous tests were performed at ambient laboratory temperature (approximately 22°C), temperature was reduced to 10°C for Test 5. Complete inactivation was achieved for all samples at both temperatures tested, thus the reduced temperature did not appear to significantly impact decontamination performance (*P* = 1.000) at the conditions tested.

### Contact time

**Fig 5:**
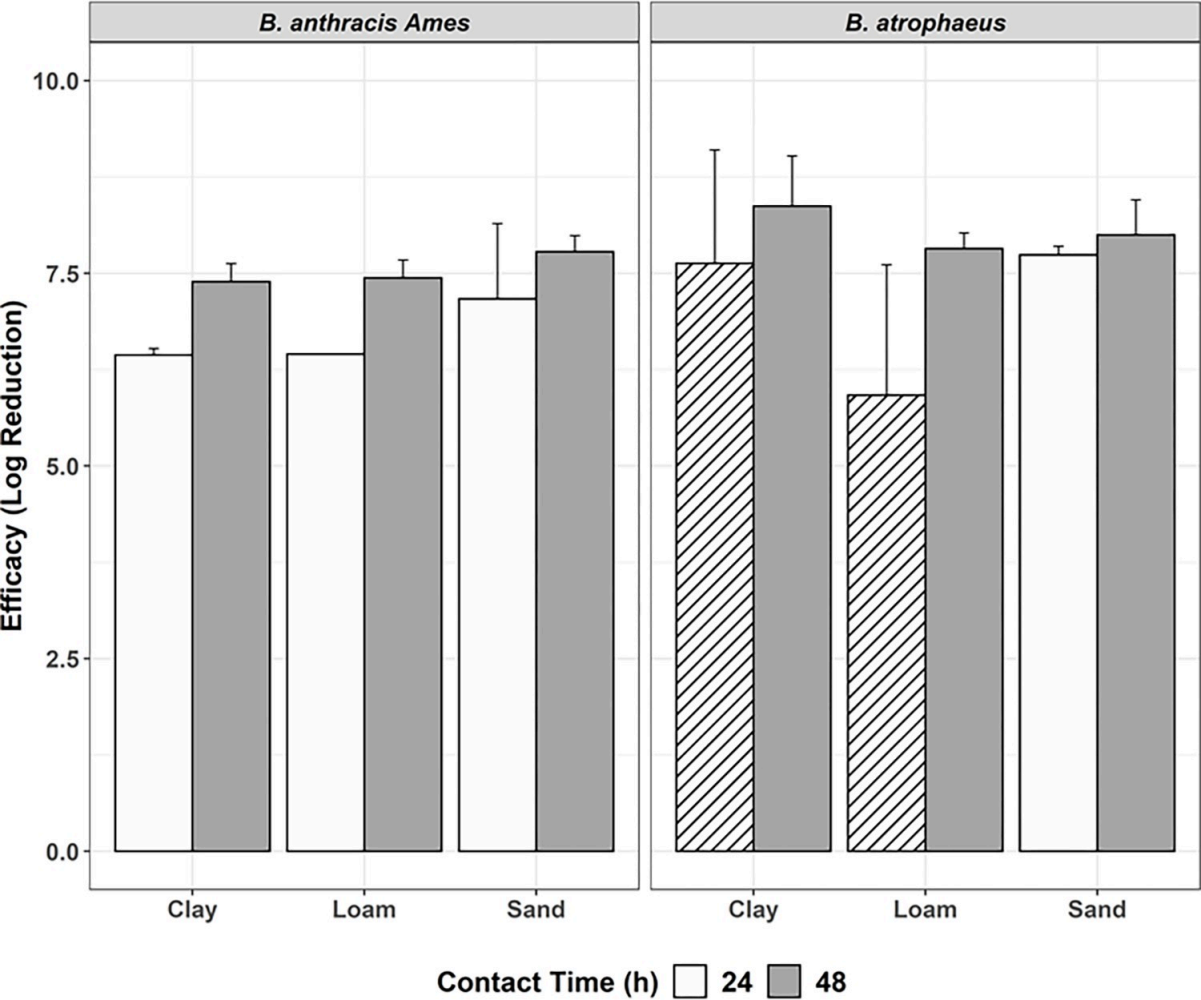
Efficacy of 2 mL of 2.5% formaldehyde, covered, at 10°C with 24-hr (white; Test 6) vs. 48-hr (gray; Test 5) contact time. Tests conducted against *B*.*a*. *and B*. *atrophaeus*, with efficacy represented by log reduction (± 95% CI) of bacteria, by soil type, and 48-hour contact time. Columns with stripes are samples that did not achieve complete inactivation.

Test 6 was conducted with the same testing parameters as Test 5, with the exception of contact time which was reduced from 48 hours to 24 hours (Fig 5). Complete inactivation was again achieved in all soils inoculated with *B*.*a*. and sandy soil inoculated with *B*. *atrophaeus*. Log reduction values were lower at a 24-hour contact time for clay and loam soil inoculated with *B*. *atrophaeus*, though decontamination was still effective with LR values of 7.69 and 5.92, respectively. Overall contact time did not have a significant effect on whether complete inactivation occurred (*P =* 0.1827).

### Soil type

All soil types were tested with both organisms for each set of testing parameters. Complete inactivation was achieved for both species in sandy soil in 5 of the 6 tests. For the clay and loam soils, complete inactivation was achieved for both species in 3 of the 6 tests. When data were separated by soil type, sample covering (lid) had a significant effect, increasing probability of complete inactivation for all three types (*P* = 0.0221, *P* = 0.039, *P* = 0.0221 for sand, clay, and loam, respectively). Formaldehyde volume was a significant effect for sandy soil, indicating a higher probability of complete inactivation when 2.8 mL formaldehyde was used (*P* = 0.0221). Refer to Table 3 for statistical analyses by soil type.

**Table 3:** Statistical analysis of ability to achieve complete inactivation, by soil type.

Soil Type	Effect	Wald Test Statistic	p-value
Clay	Cover*	4.2629	0.0390
	Concentration	1.2070	0.2719
	Contact Time	0.4573	0.4989
	Temperature	0.0000	1.0000
	Volume	2.0753	0.1497
Loam	Cover*	5.2363	0.0221
	Concentration	1.2071	0.2719
	Contact Time	1.2071	0.2719
	Temperature	0.0000	1.0000
	Volume	3.0689	0.0798
Sand	Cover*	5.2363	0.0221
	Concentration	0.0000	1.0000
	Contact Time	0.0000	1.0000
	Temperature	0.0000	1.0000
	Volume*	5.2363	0.0221

Effects noted with an asterisk (*) are statistically significant at or below the 0.05 level.

### Organism species

Both *B*.*a*. and *B*. *atrophaeus* were tested with all soil types for each test. Complete inactivation of *B*.*a*. occurred in all soil types in 5 of the 6 tests, while complete inactivation of *B*. *atrophaeus* was achieved in 3 of the 6 tests. Covering samples increased probability of complete inactivation for both *B*.*a*. and *B*.*g* (*P* = 0.0065 and *P* = 0.009, respectively). Formaldehyde volume had a significant effect on *B*.*a*. samples, with a higher probability of complete inactivation for a 2.8 mL volume of formaldehyde (*P* = 0.0065). Refer to Table 4 for statistical analyses by microorganism.

**Table 4:** Statistical analysis of ability to achieve complete inactivation, by species.

Species	Effect	Wald Test Statistic	p-value
*B*. *anthracis*	Cover*	7.4126	0.0065
	Concentration	0.0000	1.0000
	Contact Time	0.0000	1.0000
	Temperature	0.0000	1.0000
	Volume*	7.4126	0.0065
*B*. *atrophaeus*	Cover*	6.8190	0.0090
	Concentration	2.7002	0.1003
	Contact Time	1.9124	0.1667
	Temperature	0.0000	1.0000
	Volume	2.8366	0.0921

Effects noted with an asterisk (*) are statistically significant at or below the 0.05 level.

## Discussion

Decontamination of wide outdoor areas that have been contaminated with a spore forming pathogen such as *B*.*a*. will be challenging, as evidenced by the remediation efforts conducted on Gruinard Island [21]. Contamination may result from *an* intentional release, an epizootic contamination event, or environmental factors such as rain that could further spread the contamination within the affected area [22, 23]. Soil matrices remain one of the most difficult materials to effectively decontaminate, as there are many variables that can affect decontamination efficacy including pathogen type (e.g., virus, vegetative bacteria, spore-forming bacteria), environmental conditions, decontaminant chemistry, soil type, and depth of contamination within the soil column. An effective and rapid response to remediate a contaminated area following a biological contamination incident with *B*.*a*. requires foundational knowledge of a sporicides’ ability to achieve efficacy for the target pathogen within various soil types, which may affect (e.g., quenching) the decontaminant’s active ingredients. That is, liquid, oxidant-based sporicides such as bleach (chlorine- or oxygen-based), peracetic acid, and chlorine dioxide are generally ineffective in soils due to their organic matter consuming the oxidant. The one exception to this may be activated sodium persulfate [8, 18, 19].

The aim of this study was to evaluate the impact of several experimental variables on decontamination efficacy. The study matrix (Table 1) included the use of three soil types, two bacteria species, two concentrations of liquid formaldehyde solution, two volumes of formaldehyde solution applied to the samples, two contact times, two temperatures, and two sealed conditions. *B*.*a*. and *B*. *atrophaeus* spores were tested side-by-side to compare their resistance to inactivation by liquid formaldehyde in soil to further inform possible use of *B*. *atrophaeus* as a suitable surrogate organism for future testing. Additionally, this study examined the concept of covered versus uncovered conditions, which simulated the potential use of tarps in the field after in-situ application of the formaldehyde solutions to enhance decontamination efficacy. Finally, this study also examined the effect of decontamination at two temperatures: 22° and 10°C.

The results of this evaluation demonstrated the ability of liquid formaldehyde to achieve high levels of decontamination efficacy in various soil types. 2.8 mL of 5% formaldehyde (based on the amount of soil in each sample, this is equivalent to 0.35 mL formaldehyde solution per cm^3^ of soil) resulted in complete inactivation of both organisms at ambient temperature (22°C) uncovered for the duration of the 48-hour contact period. This result is consistent with efficacy achieved on Gruinard Island using this same concentration and application rate of formaldehyde [21]. To minimize the use of the liquid formaldehyde while maintaining high efficacy, both the concentration and volume were reduced to 2.5% and 2.0 mL (0.25 mL/cm^3^ soil), respectively, and evaluated for efficacy. This resulted in incomplete inactivation for both *B*.*a*. and *B*. *atrophaeus* when uncovered for the duration of the 48-hour contact period. To simulate the use of tarps in the field, this same concentration and application rate were tested with the caps placed onto the jars after the addition of the decontaminant. This modification resulted in complete inactivation of both organisms at ambient temperature after the 48-hour contact period. This enhanced decontamination effect is most likely the result of reduced loss of the liquid decontaminant due to evaporation, which would allow for a longer contact time with the formaldehyde solution at its original concentration. Additionally, the formation of formaldehyde vapor most likely occurred in the soil pores and headspace of the sample jars, potentially leading to additional inactivation.

Once the application quantity of the formaldehyde solution was optimized, the effect of a lower temperature was evaluated. Colder temperatures favor longer persistence of pathogens in the environment [24, 25] and may diminish inactivation efficacy as well [26]. The application of 0.25 mL/cm^3^ of 2.5% formaldehyde was evaluated at both 22 and 10°C targets with both temperatures resulting in complete inactivation. Since these parameters resulted in complete inactivation for both organisms, a reduced contact time of 24 hours was evaluated. This resulted in complete inactivation for *B*.*a*., but viable *B*. *atrophaeus* was recovered in both the clay and loam soil types. The reduced contact time was only tested at 10°C, therefore it is unclear if the decreased performance for clay and loam soil inoculated with *B*. *atrophaeus* was due only to the shorter contact time or a combination of contact time and temperature. A similar result was achieved when comparing 5% to 2.5% formaldehyde, where *B*.*a* was completely inactivated and *B*. *atrophaeus* was recoverable in both the clay and loam soil types.

The data presented are intended to help guide decontaminant selection to aid remediation response of soil in the event of a wide-area *B*.*a*. release. While many factors may affect decontamination efficacy of biological agents in soil, these findings demonstrate the ability to achieve complete inactivation of *B*.*a*. spores in a broad range of soil types using an application rate of 0.25 mL/cm^3^ soil of 2.5% formaldehyde and covering after application. Further research is needed to determine the minimum amount of formaldehyde (in terms of both concentration and volume) to use in soil applications while still maintaining high efficacy. Additional research is recommended on the impacts of other parameters such as temperature, and field demonstration on the use of tarps to control evaporation of formalin.

Under the Federal Insecticide, Fungicide, and Rodenticide Act (FIFRA), a pesticide must be registered by EPA before it can be legally sold or distributed in the United States. Once registered, pesticides must be used in a manner consistent with the approved label directions and claims. The results of this research do not supplant data required for product registration nor for adding additional clams to product labels. Products must be used in accordance with their label claims under FIFRA. EPA does not endorse the use of any products tested in this study. Further, this study focused on the effectiveness at inactivating the biological agent in the test matrix within a laboratory settings with appropriate health and safety controls in place. This study does not address the operational and health and safety requirements for use outside of the conditions in the laboratory in which this study was conducted.

## Supporting information

S1 Table Soil characterization. (PDF)
