# Supplementary material for: Effectiveness of formaldehyde in various soil types as a wide area decontamination approach for Bacillus anthracis spores
Source: PLoS One. 2022 Nov 18;17(11):e0277941. doi: 10.1371/journal.pone.0277941 (PMC9674150; doi:10.1371/journal.pone.0277941)
Supplement: S1 Table — (PDF) [file pone.0277941.s001.pdf]

Table S1. Soil Characterization

**Sand (Catalog #: GTS-PF)**	
Percent Sand: 90	Bulk Density (gm/cc): 1.26
Percent Silt: 4	
Percent Clay: 6	% Total Nitrogen: 0.126
USDA Textural Class: Sand	Olsen Phosphorus (ppm): 14
	Soluble Salts – 1:1 (mmhos/cm): 0.08
pH (Water): 7.1	
Buffer pH: 7.8	Base Saturation
Cation Exchange Capacity (meq/100 g): 10.6	% Potassium: 4.7
	% Calcium: 67.7
% Moisture at 1/3 Bar: 8.6	% Magnesium: 15.0
% Moisture at 15 Bar: 6.8	% Sodium: 0.5
% Organic Matter: 1.8	% Hydrogen: 12.0
**Clay (Catalog #: MCL-PF)**	
Percent Sand: 46	Bulk Density (gm/cc): 1.03
Percent Silt: 24	
Percent Clay: 30	% Total Nitrogen: 0.191
USDA Textural Class: Sandy Clay Loam	Olsen Phosphorus (ppm): 3
	Soluble Salts – 1:1 (mmhos/cm): 0.31
pH (Water): 8.0	
Buffer pH: 7.8	Base Saturation
Cation Exchange Capacity (meq/100 g): 32.6	% Potassium: 2.1
	% Calcium: 77.9
% Moisture at 1/3 Bar: 26.7	% Magnesium: 15.0
% Moisture at 15 Bar: 19.4	% Sodium: 0.4
% Organic Matter: 3.9	% Hydrogen: 4.7
**Loam (Catalog #: HCB-SL-PF)**	
Percent Sand: 40	Bulk Density (gm/cc): 0.97
Percent Silt: 34	
Percent Clay: 26	% Total Nitrogen: 0.269
USDA Textural Class: Loam	Olsen Phosphorus (ppm): 13
	Soluble Salts – 1:1 (mmhos/cm): 0.29
pH (Water): 7.8	
Buffer pH: 7.8	Base Saturation
Cation Exchange Capacity (meq/100 g): 26.6	% Potassium: 4.0
	% Calcium: 76.9
% Moisture at 1/3 Bar: 33.0	% Magnesium: 13.1
% Moisture at 15 Bar: 23.2	% Sodium: 0.3
% Organic Matter (Walkley-Black): 5.0	% Hydrogen: 5.7
